# The large-scale investigation of gene expression in *Leymus chinensis* stigmas provides a valuable resource for understanding the mechanisms of poaceae self-incompatibility

**DOI:** 10.1186/1471-2164-15-399

**Published:** 2014-05-26

**Authors:** Qingyuan Zhou, Junting Jia, Xing Huang, Xueqing Yan, Liqin Cheng, Shuangyan Chen, Xiaoxia Li, Xianjun Peng, Gongshe Liu

**Affiliations:** Key Laboratory of Plant Resources, Institute of Botany, Chinese Academy of Sciences, Nanxincun 20, Xiangshan, Beijing 100093 China; Beijing Computing Center, Beijing, China

## Abstract

**Background:**

Many Poaceae species show a gametophytic self-incompatibility (GSI) system, which is controlled by at least two independent and multiallelic loci, *S* and Z. Until currently, the gene products for *S* and *Z* were unknown. Grass SI plant stigmas discriminate between pollen grains that land on its surface and support compatible pollen tube growth and penetration into the stigma, whereas recognizing incompatible pollen and thus inhibiting pollination behaviors. *Leymus chinensis* (Trin.) Tzvel. (sheepgrass) is a Poaceae SI species. A comprehensive analysis of sheepgrass stigma transcriptome may provide valuable information for understanding the mechanism of pollen-stigma interactions and grass SI.

**Results:**

The transcript abundance profiles of mature stigmas, mature ovaries and leaves were examined using high-throughput next generation sequencing technology. A comparative transcriptomic analysis of these tissues identified 1,025 specifically or preferentially expressed genes in sheepgrass stigmas. These genes contained a significant proportion of genes predicted to function in cell-cell communication and signal transduction. We identified 111 putative transcription factors (TFs) genes and the most abundant groups were MYB, C2H2, C3H, FAR1, MADS. Comparative analysis of the sheepgrass, rice and Arabidopsis stigma-specific or preferential datasets showed broad similarities and some differences in the proportion of genes in the Gene Ontology (GO) functional categories. Potential SI candidate genes identified in other grasses were also detected in the sheepgrass stigma-specific or preferential dataset. Quantitative real-time PCR experiments validated the expression pattern of stigma preferential genes including homologous grass SI candidate genes.

**Conclusions:**

This study represents the first large-scale investigation of gene expression in the stigmas of an SI grass species. We uncovered many notable genes that are potentially involved in pollen-stigma interactions and SI mechanisms, including genes encoding receptor-like protein kinases (RLK), CBL (calcineurin B-like proteins) interacting protein kinases, calcium-dependent protein kinase, expansins, pectinesterase, peroxidases and various transcription factors. The availability of a pool of stigma-specific or preferential genes for *L. chinensis* offers an opportunity to elucidate the mechanisms of SI in Poaceae.

**Electronic supplementary material:**

The online version of this article (doi: 10.1186/1471-2164-15-399) contains supplementary material, which is available to authorized users.

## Background

Self-incompatibility (SI) is a widely distributed mechanism to prevent self-fertilization in flowering plants [1, 2]. Classic genetic studies revealed two major classes of homomorphic SI, gametophytic (GSI) and sporophytic (SSI) systems [3]. Recent studies have focused on identifying molecules that mediate SI events. In Solanaceae, Rosaceae and Plantaginaceae GSI species, the stylar inhibition of incompatible pollen is mediated through an interaction between a stylar S-RNase (female determinant) and the pollen tube-borne F-box protein, SLF [4, 5]. GSI in poppies (Papaveraceae) is mediated by a calcium-based signaling pathway that is most likely triggered by an interaction between a stigma-expressed S-glycoprotein ligand (a small extracellular signaling molecule) and its cognate plasma membrane receptor, which is a pollen-expressed Ca^2+^-channeled protein [6]. SSI in Brassicaceae is regulated by an S-haplotype-specific protein interaction between a stigma-specific S-receptor kinase (SRK, female determinant) and its cognate ligand S-locus cysteine-rich (SCR, male determinant) [7, 8].

Poaceae exhibit a GSI system controlled by at least two independent and multiallelic loci (*S* and *Z*) [9, 10]. An incompatibility reaction occurs when both the *S* and *Z* alleles of the haploid pollen are matched in the pistil. The mechanisms of the grass GSI are poorly understood relative to the well-characterized single-locus GSI system mentioned above. Numerous efforts to identify the genes of the *S* and *Z* loci have been performed, but currently, the genes for *S* or *Z* on either the pollen or pistil side remain unknown [10, 11].

Li *et al*. [12] reported the putative pollen S-gene *BM*2 identified from *Phalaris coerulescens*, which is a grass SI species. This gene was originally observed to co-segregate with the *S* genotype. Later studies indicated that *BM*2 encodes a functional thioredoxin protein, which is possibly involved in post-translational protein modification and is located at ca. 1 cM from the *S*-locus [9]. Hackauf and Wehling [13] identified a putative ubiquitin-specific protease (UBP) gene with a pistil-specific expression pattern and co-segregation with the *Z*-locus, thus suggesting the potential involvement of protein ubiquitination in the grass SI system. However, whether the UBP gene is a component of the *Z*-locus or a linked gene with suppression recombination around the *Z* locus is unknown [14]. Kakeda [15] isolated two F-box genes in *Hordeum bulbosum* based on the partial clone HAS175, which is an anther-derived marker showing complete linkage to the *S* locus. Yang *et al*. [11] constructed subtractive hybridization cDNA libraries to enrich the SI expressed genes in *Lolium perenne*.

There are certain physiological characteristics of grass SI that resemble features of the *Papaver* system and *Brassica* SSI including the dry stigma type and a rapid response to stigma-pollen interactions [14]. In these three SI systems, the stigmas critically discriminate between compatible and incompatible pollen grains and promote compatible pollen growth while preventing incompatible pollen adhesion, hydration, germination or invasion. To fulfill these unique functions, stigmas should express genes that encode the proteins required for appropriate development of and accomplishment in pollen-stigma interactions. It is likely that many of these genes are stigma-specific or preferential. To gain a better understanding of the molecular basis of stigma development and pollen-stigma interactions, it is important to generate a near-complete catalog of genes expressed specifically or preferentially in stigma cells.

Currently, genome-wide transcriptome analyses of wet, dry and semidry stigmas have been conducted on tobacco [16], Arabidopsis [17, 18], rice [19], maize [20] and *Senecio squalidus*[21]. However, stigma transcriptome data sets are not available in SI species of the Poaceae, the fourth largest family of flowering plants, which includes all cereal crops and major cultivated forage crops [22]. To develop insight into the conserved and diverse aspects of stigma development and functions across multiple species, it is useful to expand the available transcriptomic resources for incompatible species to allow comparative analysis.

*Leymus chinensis* (Trin.) Tzvel. (sheepgrass), a member of the tribe Triticeae, is a key dominant species of typical grassland communities in the Eurasian steppe region with economic and ecological value. The species is one of most important forage crops for grazing animals in China and is important in environmental protection, including soil and water conservation [23]. *L. chinensis* is a GSI species with incompatibility responses occurring on the stigma surface before or immediately after pollen germination [24].

The aims of the present study were to search for genes expressed specifically or preferentially in the stigma of the incompatible grass plant at the entire genome level using high-throughput next generation sequencing technology to perform an RNA-seq analysis on the stigmas, leaves and ovaries of *L. chinensis*. Here, the gene expression profiling of various tissues identified 1,025 genes that were predicted to be specifically or preferentially expressed in the stigma. The stigma gene sets were significantly enriched in cell-cell communication and signal transduction mechanisms, intracellular trafficking, secretion and vesicular transport. Our results also indicated that the sheepgrass stigmas shared common SI candidate genes, such as kinase genes, identified in *Lolium*. To our knowledge, this study is the first large-scale survey of gene expression in the stigmas of an SI grass species. The availability of a pool of stigma-specific or preferential genes for *L. chinensis* offers an opportunity to elucidate the molecular mechanisms of pollen-stigma interactions and SI in Poaceae.

## Results

### The identification of stigma-specific or preferential genes

**Figure 1:**
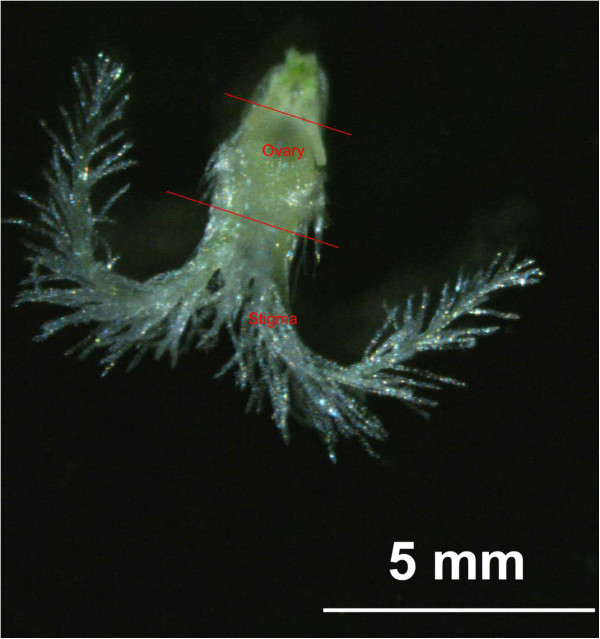
**The mature pistil that was collected just before anther dehiscence**. A long red line showing the position where a stigma was excised and a short red line showing the cutting position between ovary and rachis.

To identify the genes involved in stigma development and required for unique stigma functions, we performed an RNA-seq analysis on the stigmas, leaves and ovaries of *Leymus chinensis*. These three samples enabled us to distinguish stigma-specific transcripts from transcripts that contribute to common cellular activities and functions. Mature stigmas, which were unpollinated and reached the largest expansion, were collected from the pistils just before anther dehiscence to avoid contamination with pollen grains (Figure 1). The stigmas were excised by cutting the pistil just below the base of the plumose stigma, and the remainder of the pistil was used as an ovary sample. Approximately 1,500 stigmas, 1,500 ovaries and 20 leaves of 10-day old seedlings were collected for each RNA preparation. The mRNA from the three tissues was used to construct cDNA libraries, which were then sequenced on an Illumina HiSeq 2000 system. Paired-end 100-bp sequence reads were then generated. After quality control, 8,351,351, 6,046,077 and 5,654,202 high-quality reads were obtained from the stigmas, ovaries and leaves libraries, respectively.

**Figure 2:**
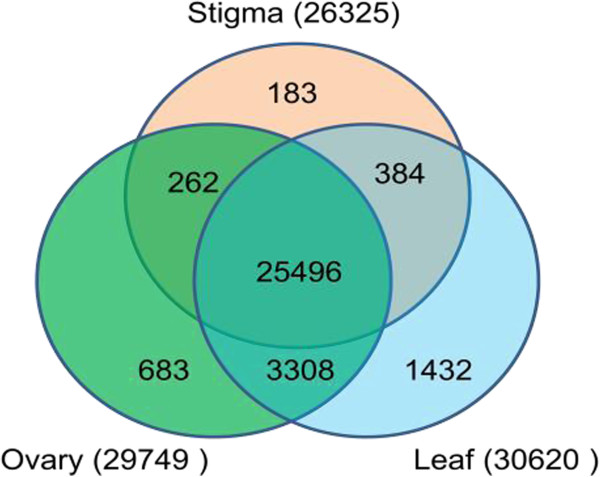
**A Venn diagram showing relationships between the three transcriptome datasets (stigmas, ovaries, and leaves)**. Numbers in parentheses are total number of expressed genes in each tissue type.

In previous studies, we performed long-read transcriptome sequencing of several cDNA libraries from different tissues of sheepgrass, including flowers before and after pollination and leaves, using a Roche 454 GS FLX sequencer [23]. A total of 87,214 unigenes were obtained, including 32,416 contigs and 54,798 singletons. The mean contig size was 607 bp. To identify the genes that corresponded to the sequencing reads in each library of the present study, the purity-filtered high-quality reads were mapped against the reference transcriptome dataset, which was generated using Roche 454 pyrosequencing technology. Altogether, 26,325, 29,748 and 30,620 contigs were detected at the stigmas, ovaries and leaves, respectively (Additional file 1). The stigma contigs, ovary contigs and leaf contigs represent about 81.2%, 91.8% and 94.4% of the total contigs of the Roche 454 sequencing dataset, respectively. Of these unigenes, 25,501 were common to all three tissues (Figure 2). A substantial number of genes showed tissue-specific expression: 183 in the stigma, 683 in the ovary, and 1,432 in the leaves.

Using a fold change of >1, false discovery rate (FDR) of <1e-05 as a cutoff to identify differentially expressed genes, 842 contigs were up-regulated in the stigma compared to the ovary and leaf (Additional file 2 and Additional file 3). These contigs were considered to be preferentially expressed in the sheepgrass stigma. Thus, 1,025 (183 plus 842) contigs were specifically or preferentially expressed in the stigma (hereafter designated the stigma dataset) (Additional file 4).

### Confirmation of stigma-specific or preferential genes by real time PCR analysis

**Figure 3:**
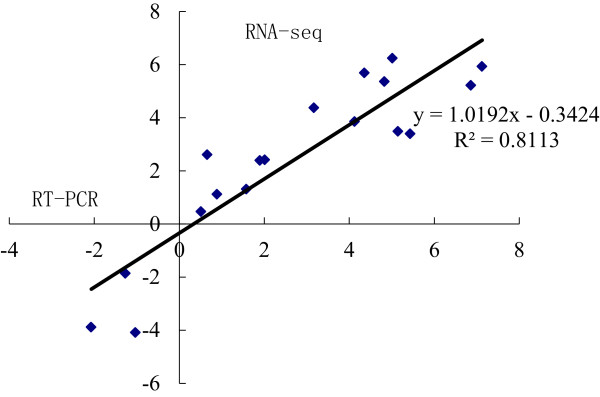
**Validation of RNA-seq results by quantitative real time RT-PCR**. Correlation plots indicating the relationship between qPCR results (fold change; Y-axis) of 18 selected genes expressed in the stigmas and leaves and the corresponding data from RNA-seq analysis (X-axis).

To validate the expression of the genes detected by the RNA-seq analysis, 18 genes with different expression patterns in the stigmas and leaves were selected for real-time PCR analysis (the primer sequences are available in Additional file 5). Scatterplots were generated by comparing the log2 fold change determined by the transcriptome analysis and real time PCR. The correlation between these two analyses was then evaluated. The results showed that the expression patterns of these genes examined by real time PCR were well correlated with those by RNA-seq (R^2^ = 0.811), thus supporting the reliability of our RNA-seq data (Figure 3).

### KOG annotation of the stigma-specific or preferential genes

**Figure 4:**
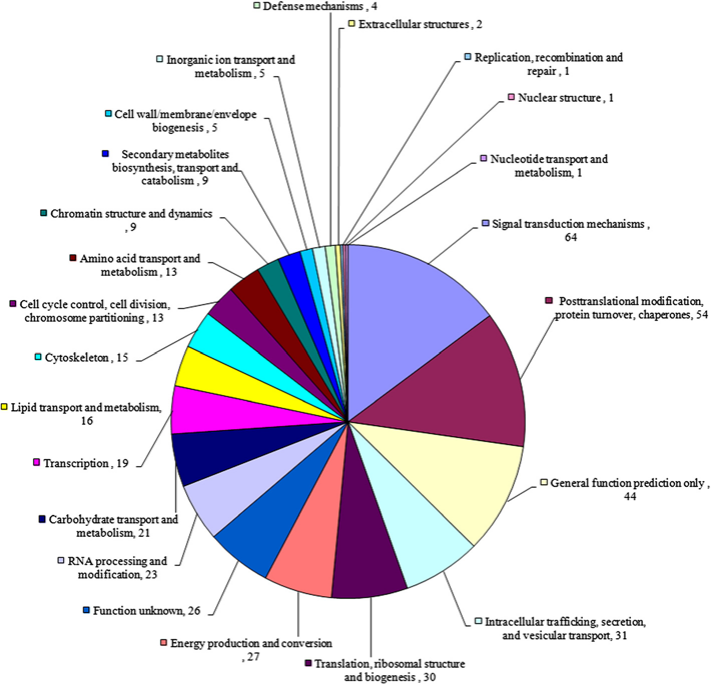
**KOG function classification**. The unigenes were aligned to the KOG database to predict and categorize possible functions. A total of 522 unigenes were assigned to 23 categories.

From the 1,025 contigs specifically or preferentially expressed in the stigma, 648 contigs had putative functions and 377 contigs were assigned as hypothetical proteins (Additional file 4) based on BLASTX similarity searches against the Swiss-Prot and NCBI non-redundant protein sequences (nr) databases. The 648 contigs were further annotated and classified based on EuKaryotic Orthologous Groups (KOG) categories. Altogether, 522 contigs were assigned KOG functional annotations (Additional file 4) and grouped into 23 functional categories (Figure 4). Among the KOG categories, “Signal transduction mechanisms” (14.8%) was the most abundant (Additional file 6).

### Gene ontology (GO) annotation

**Figure 5:**
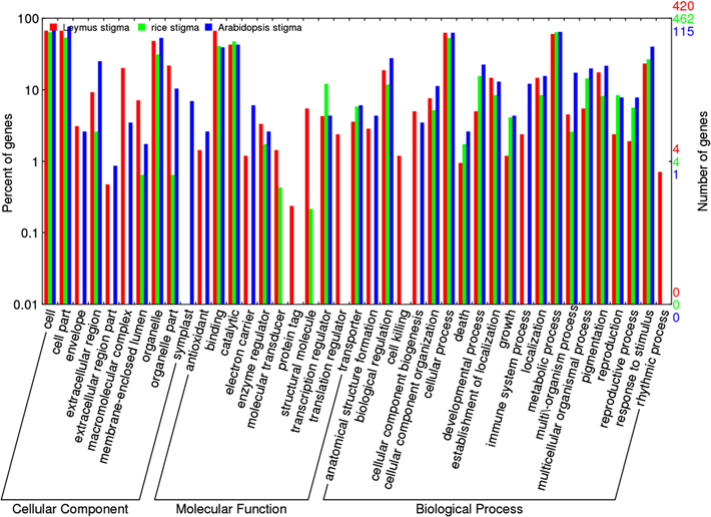
**GO assignment of all identified genes expressed specifically or preferentially in the stigmas of sheepgrass, rice and Arabidopsis**. The genes were mapped to three main categories: biological processes, cellular components and molecular functions. The right hand y-axis indicates the number of annotated genes.

Gene ontology (GO) assignment programs were utilized for the functional categorization of annotated unigenes of the sheepgrass stigma dataset. In many cases, multiple terms were assigned to the shared contig. A total of 420 contigs were categorized into 40 main functional groups belonging to 3 categories (biological processes, molecular functions, and cellular components). The GO terms of their subcategories are presented in Figure 5.

### A comparison of the GO classification of stigma-specific or preferential genes in sheepgrass, rice and arabidopsis

The stigma-specific or preferential expressed gene datasets of rice [19] and Arabidopsis [17], two compatible species, are available to provide the opportunity to compare the diversity and conservation of stigma-specific or preferential genes in species of mono- and dicotyledons. A total of 462 unigenes of rice stigmas and 115 of Arabidopsis stigmas were categorized into functional groups using the GO assignments. The WEGO online tool [25] was used to classify the GO functions for all genes of the sheepgrass, rice and Arabidopsis stigma-specific or preferential datasets to understand the distribution of gene functions at the macro level. The comparative analysis showed that these three stigma transcriptomes shared broad similarities in the proportion of genes in the three main categories and many subcategories (Figure 5). However, differences were also detected among the three species. The high proportions of genes involved in the GO terms “protein tag”, “translation regulators”, “cell killing”, and “rhythmic process” were only represented in the sheepgrass stigma dataset. The genes of the GO term “symplast” were only observed in Arabidopsis. These results suggest that despite the differences in the relationship and gross stigma morphology among these three diverse species, certain conserved mechanisms of structural development and the pollen-stigma interaction process appear to be common. Conserved stigma-specific genes may be evidence of the convergent evolution of classes of genes to acquire stigma-specific functions in diverse angiosperm species. Unique genes in the sheepgrass stigma dataset may imply intrinsic differences in stigma development and the pollination process between compatible and incompatible species.

### GO enrichment analysis

A singular enrichment analysis (SEA) [26] was performed to identify the significantly enriched GO terms in genes specifically or preferentially expressed in sheepgrass stigma. The results showed that 19 GO terms were overrepresented in the stigma based on a P-value < 0.001 and the FDR ≤ 0.05 cutoffs, which were 13, 3 and 3 for the cellular components, biological processes, molecular functions categories, respectively. The detailed results of the Go enrichment analysis are presented in Table 1.

**Table 1:** **The overrepresented functional GO terms of the sheepgrass stigma-specific or preferential genes**

GO term	Ontology	Description	No. of the elements in the whole transcriptome	No. of elements differentially expressed	No. of elements expected to be differentially	p- value	FDR
GO:0009065	P	Glutamine family amino acid catabolic process	39	7	0.651	9.80E-06	0.0078
GO:0009651	P	Response to salt stress	273	17	4.578	7.60E-06	0.0078
GO:0006970	P	Response to osmotic stress	336	18	5.641	2.80E-05	0.015
GO:0005509	F	Calcium ion binding	457	25	7.669	5.60E-07	0.00029
GO:0050897	F	Cobalt ion binding	40	7	0.6678	1.10E-05	0.0029
GO:0004857	F	Enzyme inhibitor activity	150	11	2.5158	7.90E-05	0.014
GO:0030529	C	Ribonucleoprotein complex	1072	42	17.997	6.30E-07	0.00029
GO:0005624	C	Membrane fraction	94	9	1.5779	5.30E-05	0.0043
GO:0005753	C	Mitochondrial proton-transporting ATP synthase complex	18	5	0.2982	3.20E-05	0.0043
GO:0005792	C	Microsome	55	7	0.9231	7.20E-05	0.0043
GO:0042598	C	Vesicular fraction	55	7	0.9231	7.20E-05	0.0043
GO:0005768	C	Endosome	149	11	2.5015	7.50E-05	0.0043
GO:0005626	C	Insoluble fraction	95	9	1.5947	5.80E-05	0.0043
GO:0032991	C	Macromolecular complex	3407	87	57.1998	4.80E-05	0.0043
GO:0000267	C	Cell fraction	122	9	2.048	0.00034	0.017
GO:0070013	C	intracellular organelle lumen	926	30	15.5467	0.00064	0.027
GO:0043233	C	Organelle lumen	926	30	15.5467	0.00064	0.027
GO:0031974	C	Membrane-enclosed lumen	941	30	15.7983	0.00082	0.032
GO:0000502	C	Proteasome complex	141	9	2.3671	0.0009	0.032

### Kyoto encyclopedia of genes and genomes (KEGG) pathway mapping

**Figure 6:**
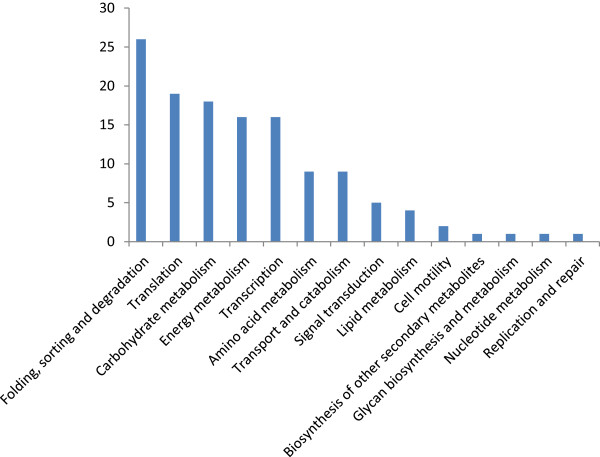
**Histogram presentation of the KEGG pathway categories**. The Y-axis indicates the number of unigenes assigned to a specific pathway. The X-axis indicates the KEGG pathway.

The genes of the sheepgrass stigma dataset were annotated based on the Kyoto Encyclopedia of Genes and Genomes (KEGG) databases [27]. The results showed that KEGG numbers could be assigned to 128 contigs (Additional file 4). These unigenes were further annotated to BRITE functional hierarchies. The BRITE functional mapping categorized the unigenes into diverse KEGG pathways (Figure 6).

### Putative transcription factors in the sheepgrass stigma dataset

**Figure 7:**
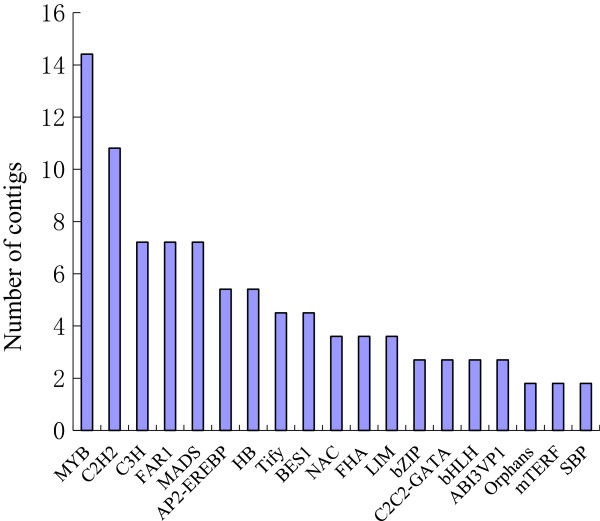
**Top 19 transcription factor families of the** ***Leymus chinensis*** **stigma dataset**. The Y-axis indicates the number of unigenes assigned to a specific TF family. The X-axis indicates the top 19 TF families.

Understanding transcription factors (TFs) patterns of expression is of particular interest because the expression and activity of these regulatory proteins is crucial for controlling the expression of numerous genes, and thus, their ability to regulate biological pathways and developmental processes. We searched the TFs in the sheepgrass stigma dataset using a BLASTX search against the PlnTFDB (Plant Transcription Factor Database) (version 3.0) (E-value ≤ 1e-10). We identified 111 putative TF unigenes from the sheepgrass stigma dataset. These unigenes were clustered into 26 TF families. The top 19 TF families in the sheepgrass stigma dataset are shown in Figure 7.

### Sheepgrass stigmas share certain potential SI candidate genes identified in other grasses

**Figure 8:**
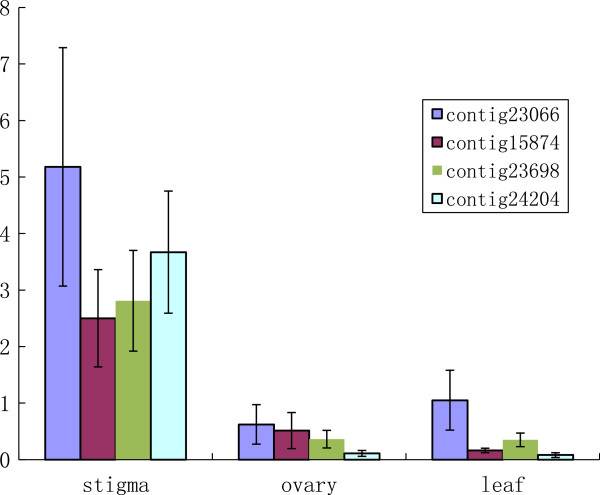
**Expression pattern of four unigenes that best hit SI candidate genes of** ***Lolium perrene***. The relative expression levels are represented in arbitrary units (A.U.) normalized to the expression level of the actin gene, used as a reference, in each sample.

Yang *et al*. [11] identified 10 expressed SI candidate genes for the *S* and *Z* loci in *L. perenne* from the SI cDNA libraries. We compared gene sequences from *Lolium* with the sheepgrass unigene set to reveal the conservation of stigma preferential genes among different SI grass stigmas. The nucleotide sequences of 10 *Lolium* SI candidate genes (Can135, accession number AM991118; Can130, AM991119; Can10, AM991120; Can136, AM991121; Can18, AM991122; Can3, AM991123; Can94, AM991124; Can4, AM991125; Can139, AM991126; and Can151, AM991127) were extracted from GenBank and blasted against the sheepgrass dataset containing 87,214 unigenes. Significantly similar sequences (using an E value ≤ 1e-05) were identified for all 10 *Lolium* SI candidate genes. Nine of the ten best hits were included in the sheepgrass stigma dataset and four (contig23066, contig15874, contig23698, and contig24204) were included in the 1,025 specific or preferential unigenes dataset (Table 2). An RT-PCR analysis confirmed the stigma-preferential expression for these four contigs (Figure 8). The *Lolium* SI candidate genes that had the best hits in the sheepgrass stigma gene dataset included all three predicted kinase genes and a kinase-partner gene of the 10 SI candidate genes, i.e., Can3 (predicted as a CBL-interacting serine/threonine-protein kinase 15), Can94 (predicted as a calcium-dependent protein kinase), Can4 (predicted as a serine/threonine-protein kinase NAK), and Can136 (predicted as a pollen-specific kinase partner protein). Two (Can3 and Can4) of the three predicted kinase genes and the kinase-partner gene showed significant similarities to the unigenes of the specific and preferential sheepgrass stigma gene set, thus indicating a potential role for kinase activity and conserved functional mechanisms in the grass SI response.

**Table 2:** **The unigenes of Leymus chinensis transcriptome dataset that best hit SI candidate genes in Lolium perenne**

Can - homology to Rice- annotation	The best hit to the Leymus chinensis unigene dataset	Identity (%)	P-value
Beta-expansin 2 precursor	contig34768 (not included in stigma)	89.81	5.00E-46
Pathogenesis-related protein PRB1-3 precursor	4-GJVU7SP04IOQ46 (in stigma)	93.52	3.00E-38
Expressed protein	contig26721 (in stigma)	91.98	2.00E-134
Pollen-specific kinase partner protein	contig23066 (included in 1025 unigene)	91.52	2.00E-125
ELMO domain-containing protein 2	contig15874 (included in 1025 unigene)	90.45	5.00E-106
CBL-interacting serine/threonine-protein kinase 15	contig23698 (included in 1025 unigene)	91.22	0
Calcium-dependent protein kinase	contig24124 (in stigma)	92.53	0
Serine/threonine-protein kinase NAK	contig24204 (included in 1025 unigene)	90.13	3.00E-177
gtk16 protein	contig15929 (in stigma)	95	1.00E-47
myb-like DNA-binding domain, SHAQKYF class	familyprotein contig 05419 (in stigma)	92.31	1.00E-36

It was proposed that *Bm*2 identified from *P. coerulescens* was involved in the SI response through the post-translational modification of other proteins by thioredoxin proteins [9]. To detect whether the *BM*2 gene was expressed in sheepgrass, we blasted the gene sequence of *BM*2 (GenBank: AF159388.1) against the sheepgrass unigene set. The result indicated that *Bm*2 matched three unigenes (contig12829, contig12830, and contig12832) with significantly similar sequences (identity > 80%, E-value ≤ 1e-10). The RT-PCR experiments validated that contig12830 was expressed in mature sheepgrass stigmas, thus suggesting that the *BM*2 gene plays an important role in the SI process.

## Discussion

The present studies have identified 1,025 genes predicted to be specifically or preferentially expressed in the stigmas of sheepgrass using high-throughput next generation sequencing technology to perform an RNA-seq analysis. This is a minimium estimate because of stringent filtering process we used in analyzing HiSeq 2000 sequencing data, incomplete detection of the expressed genes based on Roche 454 sequencing, decrease in the number of identifed genes mapped against the Roche 454 reference dataset compared to those of actually expressed genes in each tissue. It is likely that at least some of the specific or preferential sheepgrass stigma genes identified in this study function in development of the stigma papillary cells and pollen-stigma interactions. Our analysis of the stigma transcriptome demonstrates the unique features of the stigma transcriptional profile. The functional specialization of the stigma for extracellular interactions is reflected by an increased number of stigma-specific or preferential expressed genes involved in signal transduction, such as kinases, receptor-like kinases and calcineurin B-like proteins (CBL), the manufacturing of enzymes that most likely facilitate the entry of the pollen tube into the stigma, transcription factors that are possibly implicated in the pollination process, and defense-related genes that function in the defense against pathogens or potentially in response to pollination. It is hypothesized that some of these genes are potentially involved in the pollination response and SI mechanisms.

### Cell-cell communication and signal transduction

Genes predicted to function in cell-cell communication and signal transduction are particularly notable in the context of pollen-stigma interactions and SI. The sheepgrass stigma dataset contained a significant proportion of genes consistent with the primary recognition role of the stigma in discriminating genotypes of pollen grains. The most noteworthy are genes encoding various putative protein kinases, including probable receptor-like protein kinases (RLK), mitogen-activated protein kinases (MAPKs), serine/threonine-protein kinase, CBL-interacting protein kinases, and calcium-dependent protein kinase.

A stigma-specific receptor kinase, SRK, which was most extensively studied in Brassicaceae, interacts with the ligand (SCR) held on the pollen coat and initiates a signal transduction cascade that inhibits the growth of self-pollen and the pollen tube [28]. In the *Papaver* GSI mechanism, the female S-determinant (PrsS) is a small stigma-secreted signal peptideand acts as a signaling ligand. The male S-determinant (PrpS) has a small predicted extracellular domain that has been shown to be involved in both binding to PrsS and mediating SI [29]. A model for the operation of SI in *Papaver* has proposed that PrsS acts as a signaling ligand that interacts with PrpS. The sheepgrass stigma dataset contained 3 contigs, i.e., contig24113, contig28482, and contig24154, which are predicted to encode probable receptor-like protein kinase At2g42960, a probable LRR receptor-like serine/threonine-protein kinase At1g63430, and somatic embryogenesis receptor kinase 1, respectively. A homologous gene encoding receptor-like protein kinase At1g63430 was also identified in the *Senecio squalidus* SSI stigma preferential gene dataset. The contig24113, contig28482 and contig24154-encoded proteins may have a potentially important role in cell-cell communication and signal transduction in the context of pollen-stigma interactions and are therefore notable candidates for further studies of sheepgrass stigma SI mechanisms.

MAPKs are key contributors in signal transduction mechanisms and regulate important cellular processes, including cell proliferation, survival, and death in eukaryotes [30]. In addition, MAPKs are known to be involved in the activation of plant defense responses and result in programmed cell death (PCD) and pathogen resistance [30, 31]. A MAPK, p56, is responsible for mediating SI induced PCD in *Papaver*[32, 33]. p56 is involved in the loss of pollen viability, stimulation of caspase 3 like (DEVDase) activity and later DNA fragmentation in incompatible pollen [33]. We also identified two contigs, contig13945 and contig34021, in the sheepgrass stigma dataset that are predicted to encode for mitogen-activated protein kinase 10 and mitogen-activated protein kinase 1, respectively. The presence of MAPKs in the sheepgrass stigma gene dataset implies that they are likely involved in the early steps of pollination and SI responses.

Temporal and spatial changes in cytosolic Ca^2+^ concentrations generate specific Ca^2+^ signals, which are decoded by Ca^2+^ sensors [34, 35]. As a class of calcium-sensing proteins, calcineurin B-like proteins (CBLs) specifically target a group of sucrose non-fermenting-related serine/threonine kinases (SnRK3), named CBL-interacting protein kinases (CIPK), to mediate the sensed calcium signal [36–40]. The CBL-CIPK system is involved in a wide range of signaling pathways, including abiotic stress responses to drought and salt, innate immunity, plant hormone responses and K^+^ channel regulation [41, 42]. No CBLs or CIPKs have been implicated as signaling components in plant SI mechanisms. It is particularly notable that four contigs encoding CBL were observed in the analysis of the sheepgrass stigma dataset. These novel genes were not previously identified as pistil-specific or preferential in other species. It was previously mentioned that the Can3 (AM991123) gene, which putatively interacts with CBL, was identified in *L. perenne* stigma [11]. These results provide new insights into the function of CBL during pollen-pistil interactions in plants and suggest that CBLs are potentially involved in SI mechanisms.

In addition, the sheepgrass stigma dataset also contained many notable unigenes that encode calcium-related proteins, such as calmodulin-like proteins, probable calcium-binding proteins (CML49, CML13, CML18, CML16, CML22, CML21), and calcium-dependent protein kinases. The role of cytosolic free Ca^2+^ in the regulation of pollen tube growth has been well characterized [43, 44]. It has been shown that calcium signaling is involved in the self-incompatibility response of *Papaver* and *Brassica*[45, 46]. In analogy to *Papaver* and *Brassica* SI, there is some evidence for the involvement of Ca^2+^-mediated signaling in grass SI. The application of Ca^2+^ antagonists to isolated stigmas resulted in the inhibition or delay of the SI response on self-pollination in *S. cereale* and *L. perenne*[10, 47]. It is tempting to hypothesize that stigma S and Z determinants act as signal molecules in grass by interacting with the “self” pollen S and Z partners at the tube plasma membrane, thus inducing a Ca^2+^-mediated signal cascade that results in pollen tube inhibition. However, whether a Ca^2+^-mediated signaling cascade is involved in grass SI is unknown.

Many recent studies have also implicated ROS/H_2_O_2_ as signaling molecules involved in plant reproductive processes such as pollen tube growth [48–50] and pollen–stigma interactions [51, 52]. Because peroxidases can generate and consume H_2_O_2_[53], they should be considered as potentially important components of signal transduction pathways. *Senecio squalidus* stigmas accumulate high amounts of ROS, particularly H_2_O_2_, in their epidermal cells (papillae). The first plant peroxidase gene, *SSP* (stigma-specific peroxidase), expressed specifically in stigma papillae was identified in *S. squalidus*[54]. In the sheepgrass stigma dataset, we detected two contigs that encode peroxidase. The potential signaling role of these interesting genes deserves further investigation.

### Enzymes most likely facilitating entry of the pollen tube into the stigma

In flowering plants with dry stigmas, the stigmatic surface is covered by a continuous layer of cuticle, which is a significant barrier to pollen tube entry [55]. For successful pollination and subsequent fertilization, this barrier must be breached by cutinases from the pollen grain surface and stigma pellicle [56].

Cutinase is an esterase that degrades cutin [57–59]. Active cutinases have been detected in the pollen of *Brassica*[60] and *Tropaeolum*[61], in which they predominantly localize to the intine region of the pollen wall. Knox *et al*. [62] has suggested that esterases from the pollen and stigmatic pellicle combine to form an “active cutinase complex” which degrades the cuticle in the region of penetration. Although cutinases have been implicated in the process of pollen tube penetration in many species with dry stigmas, none have been fully characterized at the molecular level [63]. The identification of the genes that encode plant cutinases is important for elucidating the digestion mechanism of the stigmatic cuticle during pollen–stigma interactions. The sheepgrass stigma dataset contains three contigs that were predicted to encode GDSL esterase, two contigs for probable pectin esterase, and four contigs for pectin esterase inhibitor. These unigenes are very interesting candidates for further studies on enzymatic degradation of the stigmatic cuticle during pollination.

When the cuticle has been breached, pollen tubes invade the stigmatic papillary cell wall by growing between its outer and inner layers. The pollen tube then continues to grow through the intercellular spaces (extracellular matrix, ECM) of the transmitting tissue towards the ovary [64, 65]. Much evidence has indicated that pollen- and stigma-derived enzymes and proteins most likely facilitate the entry of the pollen tube into the stigma by loosening the papillary cell wall and the ECM and/or modifying the growing pollen tube wall. These enzymes and proteins included expansins [66], pectin esterases [67], polygalacturonases [68], glucanases [69], xylanases [70], pectate lyases, pectin methylesterases [67], and peroxidases [71]. Lipid transfer proteins (LTP), one of the most abundant proteins present in the stigma of several species, have cell wall-loosening activity that is similar to expansion, despite its assumed role in lipid metabolism.

The sheepgrass stigma dataset contained many unigenes for putative cell wall-localized proteins that might function in regulating the expansion or loosening of the stigmatic papillary cell wall and ECM, thus regulating the entry of the pollen tube into the stigma. Among these proteins are two expansins, five pectinesterase, a pectin esterase inhibitor, two peroxidases, five polygalacturonases, three pectate lyases, and seven lipid transfer proteins. Because their better studied relatives exhibit cell wall-loosening activity and have been implicated in ECM remodeling and the disassembly of ECM structural components, some of these proteins (e.g., expansins and hydrolase) may facilitate the penetration of the pollen tube into the stigma by modifying the papillary cell wall and ECM.

Expansins are cell wall-loosening proteins that regulate cell wall expansion and cell enlargement in a pH-dependent manner [72]. The expansin superfamily (EXP) comprises four distinct families: expansin A (EXPA), expansin B (EXPB), expansin-like A (EXLA) and expansin-like B (EXLB) [73]. Experimental evidence has indicated that EXPA and EXPB proteins function in the loosening and extension of cell walls, whereas the exact functions of EXLA and EXLB remain unknown [74, 75]. The sheepgrass stigma dataset contained A and B expansins. The latter have been suggested to act as cell wall-loosening agents to facilitate pollen adhesion and pistil penetration [76]. Yang *et al*. [11] also identified a prominent SI candidate, Can135, representing a putative EXPB protein in *L. perenne* stigmas. Because the assumed functions of expansins have been implicated in the process of pollen tube penetration, it is possible that the expansins expressed preferentially in sheepgrass stigmas might be related to pollen-stigma interactions and SI.

### Transcription factors possibly implicated in the pollination process

The processes underlying pollen recognition and pollen tube growth require the concerted action of genes that are regulated by transcription factors [77]. A total of 111 unigenes encoding the transcription factors of 26 families were identified in mature sheepgrass stigmas. Most TFs (58%) belonged to seven families: MYB, C2H2, C3H, FAR1, MADS, AP2-EREBP, and HB. Among these families, the MYB genes form the largest category. Most plant MYB genes encode proteins of the R2R3-MYB class [78]. Numerous R2R3-MYB proteins are involved in the control of plant-specific processes including: primary and secondary metabolism, cell fate and identity, developmental processes, and responses to biotic and abiotic stresses [79, 80]. For example, several R2R3-MYB proteins encoded by AtMYB0/GL1, AtMYB23 and AtMYB66/WER are involved in the determination of epidermal cell type. AtMYB0 and AtMYB23 control trichome initiation in shoots, whereas AtMYB66 controls root hair patterning [81]. Two closely related R2R3-MYBs, AtMYB88 and AtMYB124/FLP regulate stomatal differentiation and patterning. AtMYB98 regulates synergid cell differentiation during female gametophyte development, pollen tube guidance and the formation of the filiform apparatus [82]. Can151, which is an important SI candidate gene that represents a putative Myb-like protein, was identified in *L. perenne* stigmas [11]. MYB transcription factors were also detected in the stigmas of many other species, such as Arabidopsis [17, 18], rice [19], tobacco [16], maize [20], and crocus [83]. Similar to our studies, the transcriptome analysis of mature maize stigmas also indicated that the MYB genes represented the largest category of transcription factors. The preferential expression of MYB family genes in the mature or pollinated stigma of many species implicates its possible role in the pollination process and pollen-stigma interactions. Recently, Gómez-Gómez *et al*. [84] isolated *CsMYB1*, which is a transcription factor belonging to the R2R3 family gene, from *Crocus sativus. CsMYB1* is highly expressed in stigma tissues and poorly expressed in tepals, whereas no transcript was detected in either anthers or leaves. *CsMYB1*’s expression is developmentally regulated with no transcript detected in early stage stigmas but high levels occurring in later stages. It was assumed that CsMYB1 is possibly involved in the control of stigma morphological development. Although MYB genes have been identified and isolated in the stigma of many species, the precise functions of these ubiquitous stigma TFs in the pollination process are uncharacterized.

### Defense-related genes

The stigmas of flowering plants are stubbornly resistant to pathogen invasion. Both wet and dry stigmas possess an effective pathogen defense system [1] and express a wide range of pathogenesis-related genes that presumably contribute to this stigmatic defense system [17, 18, 85]. These defense-related genes might function not only in defense against pathogens, but also in response to pollination. The mechanisms originally evolved in defense against pathogens may have been recruited to identify and reject self-pollen in an SI response [1]. The evidence indicated many striking similarities in the process of epidermal cell penetration by fungal spores and the pollen tube, including parallels in cell wall modification, the presence of phenolic derivatives, the accumulation of high levels of extracellular calcium, and the synthesis of β1-3 glucan callose [1, 19].

Our transcriptome analyses have identified several stigma-specific or preferential genes that encode proteins potentially involved in defense responses, such as dihydroflavonol-4-reductase (DFR) and Bax inhibitor 1 (BI-1). DFR are key regulatory enzymes of flavonoid biosynthesis [86]. Flavonoids are ubiquitous plant secondary metabolites and are accumulated in different organs and tissues of plants [87]. Flavonoids are mainly involved in protecting plants against predation and pathogens (bacteria and fungi) [88]. High amounts of stigmatic flavonoids may be toxic to potential pathogens but are not toxic to pollen. It can be speculated that an unknown signaling mechanism identifies a pollen grain as a pollen grain and not a fungal spore or bacteria.

Plant cells can respond to various stimuli, including fungal toxins and biotic and abiotic stresses, by initiating programmed cell death. Bax inhibitor 1 proteins act as negative regulators of stress-related programmed cell death in plants and animals [89]. The stable overexpression of this cell death inhibitor in barley reduces susceptibility to the necrotrophic fungus *Fusarium graminearum*[90]. The transient or stable overexpression of BI-1 strongly supports the penetration of *Blumeria graminis* f. sp. *hordei* into barley epidermal cells [90, 91]. Here, we present the first report that genes encoding BI-1 proteins were expressed in the stigma of flowering plants. However, further analysis is necessary to better understand the involvement of these notable proteins in pathogen defense and pollen-stigma interactions.

## Conclusion

For the first time, we present a large-scale investigation of gene expression in the stigmas of sheepgrass (*L. chinensis*), a Poaceae GSI species, using a high-throughput RNA-seq analysis. Altogether, 26,325 contigs were detected in the stigmas. Of these contigs, 1,025 showed specific or preferential expression. Quantitative real-time PCR confirmed the expression patterns of the genes examined by RNA-seq. This large-scale survey represents an efficient approach to identify the genes involved in the stigma pollination process. The functional specialization of the stigma for pollination behavior is reflected by unique features of its transcriptional profile. Many identified stigma-specific or preferential genes were potentially involved in encoding signaling molecules, such as protein kinases and receptor-like kinases, proteins and enzymes modifying the cell wall, cuticle or ECM to facilitate the entry of the pollen tube into the stigma, and defense-related proteins that function to defend against pathogens or in response to pollination. Most of the genes identified by the RNA-seq analysis were not previously studied or reported to be expressed in stigmas, and their potential involvement in stigma development and pollen-stigma interactions opens new avenues to better understand the grass GSI system in terms of function and evolution. The future functional characterization of these genes promises to elucidate the mechanisms that underlie incompatible pollen-stigma interactions in grass GSI systems.

## Methods

### Plant materials, RNA extraction, library construction and illumina sequencing

The plant materials were harvested from sheepgrass plants grown under natural conditions in a field of the Beijing Botanical Garden, Chinese Academy of Sciences, Beijing, China. The stigmas were collected 1–10 hours before floret flowering by cutting the pistil below the base of the stigma, and the remainder of the pistil was used for the ovary samples. The leaves were collected from 10-day-old seedlings.

The total RNA from the mature stigmas, mature ovaries and leaves was extracted using TRIzol Reagent according to the manufacturer’s instructions (Invitrogen, Carlsbad, CA, USA). The quality of the total RNA was determined using a NanoDrop 2000 (Thermo Fisher, USA). The mRNA was purified from the total RNA samples using a Dynabead mRNA Purification Kit according to the manufacturer’s instructions (Invitrogen, Carlsbad, CA, USA), and the quality was assessed using an Aligent 2100 Bioanalyzer (Agilent Technologies, Inc., Waldbronn, Germany). Double-stranded cDNA was synthesized using the SuperScript Double-Stranded cDNA Synthesis Kit (Invitrogen, Carlsbad, CA, USA). Specific adapters were ligated to the fragmented cDNA and denatured to generate single-stranded cDNA followed by emulsion PCR amplification. The sequencing was performed using an Illumina HiSeq 2000 sequence analyzer at Hanyu Genomics Institute (Shanghai, China).

The sequence reads generated from each tissue sample were aligned to the reference transcriptome dataset obtained by Roche 454 pyrosequencing technology using SOAP2 software [92]. For the specific mapped unigenes with uniquely matched reads, the transcript abundance level was normalized using the RPKM (Reads Per kb per Million reads) method. The RPKM values were computed as proposed by Mortazavi *et al*. [93]. If there was more than one transcript for a gene, the longest one was used to calculate its transcript abundance and coverage.

### Real-time PCR analysis

The total RNA (4 μg) was reverse-transcribed with an oligo (dT) primer for cDNA synthesis using a SuperScript III First-Strand Synthesis Kit (Invitrogen). Gene-specific primers were designed using PRIMEREXPRESS software (Applied Biosystems). The primer sequences are listed in Additional file 5. Quantitative PCR assays were performed in triplicate using SYBR Green Real-time PCR Master Mix (Toyobo, Osaka, Japan) with a Bio-Rad CFX96 Real-Time Detection System. The quantitative variation in the different replicates was calculated using the delta-delta threshold cycle relative quantification method. Amplification of *L. chinensis* actin was used as an internal control to normalize all data.

### Annotation, functional classification and pathway analysis

All mapped contigs were annotated with GetORF from the EMBOSS package [94]. The ORF of each predicted protein was used for BLASTP searches against the Swiss-Prot and NCBI nr databases with thresholds of E-value ≤ 1e-5. Domain-based alignments were performed against the KOG database at NCBI with a cut-off E-value of ≤1e-5. GO annotations for describing the biological processes, molecular functions, and cellular components were analyzed by GoPipe using a BLASTP search against the Swiss-Prot and TrEMBL databases with an E-value ≤ 1e-5 [95]. The WEGO online tool [25] was used to classify the GO functions for all genes of sheepgrass, rice and Arabidopsis stigma-specific/preferential datasets to understand the distribution of gene functions at the macro level. Another GO analysis tool, SEA [26], was used to identify overrepresented GO terms. KEGG pathways annotations were performed using the KEGG Automatic Annotation Server (KAAS) with the bi-directional best-hit information method [27]. KAAS annotates every submitted sequence with KEGG orthology (KO) identifiers that represent an orthologous group of genes directly linked to an object in the KEGG pathways and BRITE functional hierarchy [27, 96]. To identify transcription factor families represented in the sheepgrass stigma dataset, unigene sequences were searched against a complete list of transcription factor protein sequences of the Plant Transcription Factor Database (PlnTFDB: http://plntfdb.bio.uni-potsdam.de/v3.0/downloads.php) using BLASTX with an E-value cutoff of ≤ 1e-10.

### Availability of supporting data

Sequence data from this article were deposited in the NCBI Sequence Read Archive (SRA065691).

## Electronic supplementary material

Additional file 1: **Genes expressed in the stigmas, leaves, and ovaries of** ***Leymus chinensis***. RPKM: Reads Per Kilo bases per Million reads; NA: Not applicable. (XLS 6 MB)

Additional file 2: **Comparison of gene expression between the stigmas and leaves of** ***Leymus chinensis***. (XLS 5 MB)

Additional file 3: **Comparison of gene expression between the stigmas and ovaries of** ***Leymus chinensis***. (XLS 5 MB)

Additional file 4: **1,025 genes specifically or preferentially expressed in the stigma of** ***Leymus chinensis***. (XLS 847 KB)

Additional file 5: **A list of real- time PCR primers used in the present study**. (XLS 24 KB)

Additional file 6: **The** ***Leymus chinenesis*** **stigma specifically or preferentially expressed genes predicted to function in signal transduction mechanisms**. (XLS 24 KB)
